# The Impact of Comorbid Depression on Adherence to Therapy for Multiple Sclerosis

**DOI:** 10.1155/2011/271321

**Published:** 2011-08-02

**Authors:** M. Tarrants, M. Oleen-Burkey, J. Castelli-Haley, M. J. Lage

**Affiliations:** ^1^Medical Affairs, Teva Pharmaceuticals, 901 East 104th Street, Suite 900, Kansas City, MO 64131, USA; ^2^HealthMetrics Outcomes Research, 120 Anchorage Circle, Groton, CT 06340, USA

## Abstract

*Objective*. Examine the impact of comorbid depression on adherence to disease-modifying therapy (DMT) for multiple sclerosis (MS). *Methods*. A retrospective database was used to identify patients with MS treated with a DMT. Patients with MS and comorbid depression were matched to patients with MS only. Adherence to DMT was proxied by the medication possession ratio (MPR) and multivariate regressions were used to examine the association between comorbid depression and adherence to DMT. *Results*. Patients with comorbid depression had a 10 point lower MPR (*P* < 0.01) and were less likely to achieve a MPR of at least 80% (odds ratio (OR) = 0.55; 95% confidence interval (CI) 0.42–0.74) than those without depression. While treatment with an antidepressant generally had no significant impact on the likelihood of achieving an MPR threshold of 80% (OR = 1.32; 95% CI 0.50–3.48), adherence to antidepressant therapy guidelines were associated with improved adherence to DMT therapy. *Conclusions*. MS patients with comorbid depression were approximately half as likely to be adherent to their DMT relative to patients with MS without depression. Although treatment with antidepressant therapy generally did not improve the likelihood of adherence, treatment with antidepressants for at least 6 months was associated with better adherence to DMT.

## 1. Introduction

Multiple sclerosis (MS) is the most common disabling neurological condition for young adults and adolescents in the United States [1]. Approximately 400,000 Americans have MS, and every week about 200 people are diagnosed with the disease [2]. While the results of the existing burden-of-illness studies conducted for MS in the United States vary according to the data sources used [3], they all report substantial economic impacts of MS, both to the individual and to the nation. The cost of each MS relapse has been reported to be approximately $12,870 [4]. The average cost of MS for the individual patient, each year, is anywhere from $12,879 (2004 USD) [5] to $34,000 (1994 USD) [6]. These figures translate to a national cost of $5.2 billion ($12,879 × 400,000 Americans diagnosed with MS) [5] to $6.8 billion (based on prevalence figures used by the authors of [6], annually. 

While MS is incurable, Food and Drug Administration- (FDA-) approved disease-modifying therapies (DMTs) have been shown to reduce the rate of relapse and to slow disease progression [7, 8]. However, medication can only work if it is taken. Lack of medication adherence has been shown to be associated with increased patient morbidity, poorer quality of life, and increased financial strain on health care institutions [9]. In addition, research has also shown that adherence is generally suboptimal among patients with MS [10–13]. 

One factor that has been shown to negatively impact adherence among patients with MS is the presence of comorbid depression [10, 13]. This factor may affect a large portion of the MS population, since depression is common among individuals with MS. Research has shown that up to one-half of all MS patients [14, 15] have comorbid depression, and that the prevalence of this comorbidity is three-times the rate of major depression and psychiatric comorbidity in community-based samples and greater than the rate of depression among patients with other neurological disorders [16]. 

Given the potential impact of depression on MS adherence as well as the relatively large prevalence of this comorbidity, this analysis was designed to quantify the impact of comorbid depression and antidepressant therapy on adherence to disease-modifying therapy (DMT) for MS. Given the previous literature reporting a connection between mood disorders and adherence among patients with MS [10], we hypothesized that a diagnosis of comorbid depression would have a negative impact on adherence. In addition, we examined the impact of receipt of antidepressant therapy on adherence to MS adherence. In so doing, the analysis quantifies the association between comorbid depression, receipt of antidepressants, and adherence to MS therapy.

## 2. Materials and Methods

### 2.1. Data

The MedStat MarketScan Commercial Claims and Encounters (CCAE) and Medicare (MDCR) databases provided the data for these analyses, which focused exclusively on data from January 1, 2004 through March 25, 2009. These retrospective insurance claims databases include private sector health data from approximately 100 payers and contain data on clinical utilization, expenditures, and enrollment across inpatient, outpatient, prescription drug, and carve-out services. The deidentified databases are fully Health Insurance Portability and Accountability Act (HIPAA) compliant and link paid claims and encounter data to detailed patient information across sites and types of providers over time.

### 2.2. Sample

**Figure 1:**
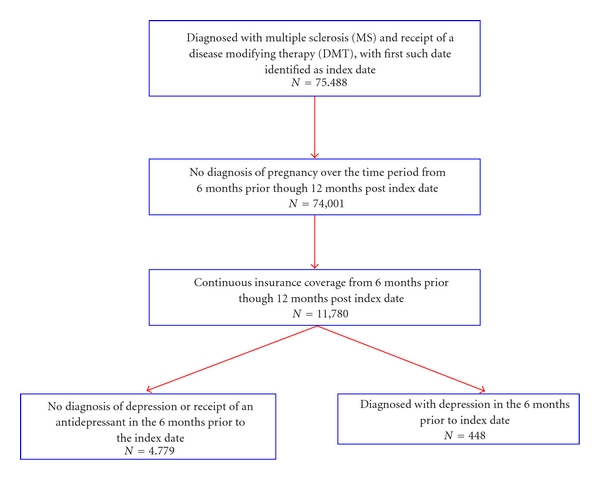
Inclusion/exclusion criteria.

To be considered for inclusion in the study, patients had to have received at least one diagnosis of MS (ICD-9-CM code of 340.xx) and a disease modifying therapy (DMT), with the first such receipt in the date window identified as the index date and the initial DMT received identified as the intent-to-treat (ITT) medication. Patients were excluded if they were diagnosed with pregnancy (ICD-9-CM code of 630.xx-676.xx, V22.2x, or V22.3x) over the time period from 6 months prior to the index date (i.e., the pre-period) through 12 months after index date (i.e., the postperiod). Finally, patients were required to have continuous insurance coverage from the start of the pre-period through the end of the post-period. These criteria resulted in a sample of 11,780 individuals (Figure 1).

Given the above cohort of individuals, the group was then subdivided into patients who had neither a diagnosis of depression nor the receipt of an antidepressant in the pre-period (i.e., MS Only) and those who were identified with comorbid depression (i.e., MS & Depression). To be identified as having depression in the 6 month pre-period, patients were required to receive 2 or more outpatient or 1 or more inpatient diagnoses of depression (ICD-9-CM of 296.2x, 296.3x, or 311.xx), or 1 or more diagnoses of depression and 1 or more filled prescriptions for an antidepressant. For patients who did not receive an antidepressant, the requirement of one inpatient diagnosis or two outpatient diagnoses of depression was utilized to insure that a patient was diagnosed with depression and not simply screened for depression. Furthermore, such an algorithm is commonly used to identify diagnoses in retrospective databases [17, 18]. There were 4,479 individuals diagnosed with MS who had no evidence of depression (MS Only) and 448 individuals identified as having MS and comorbid depression (MS & Depression). Of the 448 individuals with MS and Depression, 379 individuals received at least one prescription for an antidepressant.

Given the above sample of patients, three subgroups of patients were examined. One group consisted of a matched group of patients with MS only to those with MS and depression, where patients were matched 1 : 1 without replacement based upon age, sex, region of residence, year of index date, and intent-to-treat medication. This matching resulted in a successful match rate of 97% and a final sample of 876 individuals: 436 individuals with MS and depression and 436 individuals with MS only. In addition to examining patients with MS only to those with MS and depression, we also examined adherence by comparing patients who were diagnosed with depression but did not receive an antidepressant (*N* = 59) to those diagnosed with depression who did receive an antidepressant (*N* = 359). These two groups were matched 1 : 1 without replacement using the same factors used to match the MS Only group to the MS & Depression group. This process resulted in a final sample of 76 individuals: 38 individuals with a diagnosis of depression and no receipt of an antidepressant and 38 individuals who were both diagnosed with depression and filled at least one prescription for an antidepressant. The third group compared patients who received an antidepressant based upon adherence to treatment guidelines for use of antidepressants [19]. Specifically, patients were divided based upon receipt of at least a 6-month supply of antidepressants. Again, the two cohorts were matched 1 : 1 without replacement, with a final sample of 142 individuals: 71 treated according to guidelines, and 71 who received less than 6 months of antidepressants.

### 2.3. Measuring Adherence

While adherence generally measures the extent to which a patient acts in accordance with the prescribed interval and dose of a dosing regimen [20], such information is not typically available in a retrospective claims database. As such, adherence is “...operationalized in retrospective assessments as the number of doses dispensed in relation to the dispensing period, often called the medication possession ratio (MPR)” [20]. This variable is measured as the percentage of days the ITT medication was available to the patient based on prescription fill dates over the 1-year post-period, and is a common proxy for adherence when examining retrospective data [6, 21, 22]. As a test of the robustness of results, we also considered an alternative proxy for adherence. Specifically, for each patient the variable proportion of days covered (PDC) was also constructed [23]. The PDC, like the MPR, is a measure of the percentage of days a person received their ITT drug over the year after index date. However, in contrast to the MPR, the PDC does not allow for prescriptions to overlap. As a result, the PDC, unlike the MPR, will always have an upper bound of 1. The results reported here are not sensitive to this alternative specification and hence, results where PDC was used as the dependent variable were not reported. It should be noted, however, that neither the MPR nor PDC can measure whether an individual took a prescription once filled, whether an individual filled a prescription that was written by their physician, or whether the individual stopped their ITT medication on or against physician advice. 

### 2.4. Statistical Analyses

Descriptive statistics were compared between groups using chi-square statistics. Multivariate regressions were used to examine the impact of comorbid depression as well as medical treatment for depression on the likelihood of a patient being adherent to their MS ITT DMT. Specifically, ordinary least squares regressions were used to examine the impact of depression on MPR when MPR was measured as a continuous variables, while logistic models were used to examine the association of depression on the probability of achieving an MPR threshold of at least 80% [24, 25]. 

In multivariate models it was hypothesized that, in addition to the factors that groups were matched on (age, sex, region of residence, ITT DMT therapy, and year of index date), other patient characteristics might also affect adherence. Specifically, these analyses also examined the association between patient insurance status, characteristics of ITT medication, patient general health, and patient disability level and adherence to ITT DMT. Characteristics of the ITT medication that were examined were the amount of copayment the individual was responsible for as well as whether the ITT medication was delivered via mail order. It was hypothesized that patients required to make a copayment for their medication may be less adherent to their medication regimen while mail order delivery may be associated with ease of use, and therefore may be associated with improved adherence. Patient general health status was proxied by the Charlson Comorbidity Index (CCI) [26, 27]. This measure, which is well validated as an algorithm for predicting hospital mortality and is often used in retrospective database analyses, is constructed of a weighted average based upon the receipt of diagnostic codes associated with 17 medical conditions. Examples of conditions included in the CCI include renal disease, myocardial infarction, malignancy, liver disease, cerebrovascular disease, congestive heart failure, dementia, and diabetes [28]. Finally, patient level of disability was proxied by the presence of musculoskeletal or medical disabilities. These disabilities were proxied by examining the presence of diagnoses for secondary conditions associated with muculoskeletal or medical disabilities, and prior research has shown that these proxies are associated with both the presence and severity of disabilities [29]. 

All analyses were conducted using SAS version 9.3. Findings of *P* values ≤ 0.05 were considered to indicate statistically significant results.

## 3. Results and Discussion

### 3.1. Results: Descriptive Statistics

As expected, in all cases, groups were identical in terms of the matching variables age, sex, region of residence, ITT DMT, and year of index date. When comparing the MS only group to patients with MS and depression (Table 1), results revealed that patients diagnosed with comorbid depression were significantly less likely to be insured via point-of-service insurance (13.30% versus 22.71%; *P* < 0.01), but significantly more likely to be insured via a preferred provider organization (51.83% versus 43.81%; *P* = 0.02) or to have “other” insurance (4.36% versus 1.83%; *P* = 0.03). In addition, patients with comorbid depression were less likely to have a copayment associated with their index ITT medication prescription (77.98% versus 84.86%; *P* = 0.01). Comparing patients with MS only to those with MS and depression revealed significant differences in both general health and functional limitations between the two groups, with patients with comorbid depression generally in poorer health. For example, patients diagnosed with comorbid depression were less likely to have a Charlson score of 0 (62.18% versus 75.92%; *P* < 0.01) and more likely to have a Charlson score of 1-2 (30.28% versus 21.33%; *P* < 0.01) or 3 or more (7.57% versus 2.75%; *P* < 0.01). Similarly, patients with comorbid depression were significantly more likely to be diagnosed with a musculoskeletal limitation (60.09% versus 41.51%; *P* < 0.01) or medical limitation (48.17% versus 31.42%; *P* < 0.01). 

In contrast to the comparison of patients with MS Only to those with MS and depression, comparisons between patients with MS and comorbid depression treated and untreated with antidepressants revealed few differences. For example, Table 2 compared patients diagnosed with depression who did not receive an antidepressant to those patients who were both diagnosed with depression and received at least one prescription for an antidepressant. There were no statistically significant differences found among these two groups when comparing insurance status, copayment or mail order status, CCI, or disability proxies (Table 2). Similarly, comparing patients who received at least 6 months of an antidepressant to individuals who received less than 6 months of an antidepressant (Table 3) revealed few differences between the two groups.

### 3.2. Results: Ordinary Least Squares Regressions

Table 4 shows the results of the ordinary least squares regression models that examine the association between comorbid depression and patient adherence to ITT DMT for MS. Comparing patients with Ms only to those with MS and depression, results indicate that, after controlling for patient insurance type, characteristics of initial DMT dispensed, patient general health, and functional limitations, patients with comorbid depression had a 10% lower MPR score than those without comorbid depression (coefficient = −0.10; *P* < 0.01). Results also reveal that patients with health-maintenance insurance, compared to those with a preferred provider organization had significantly lower medication adherence (coefficient = −0.10; *P* = 0.02) while patients who had a copayment associated with their ITT DMT had significantly higher medication adherence (coefficient = 0.06; *P* = 0.05). 

Comparing patients diagnosed with depression but untreated to those both diagnosed and treated with an antidepressant revealed no significant association between treatment for depression with antidepressants and patient adherence to their MS therapy (coefficient = 0.07; *P* = 0.52). However, patients treated with an antidepressant for at least 6 months compared to those treated for less than 6 months have, on average, a 12-point higher MPR (coefficient = 0.12; *P* = 0.05) after controlling for insurance type, characteristics of initial DMT dispensed, and patient general health and functional limitation.

### 3.3. Results: Logistic Models

**Figure 2:**
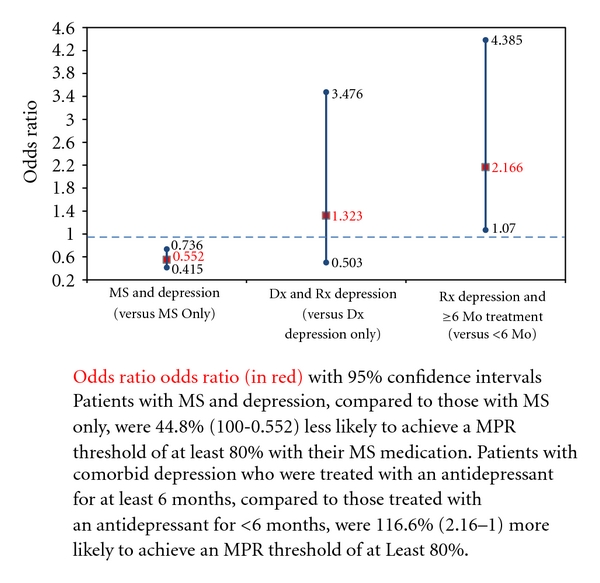
Impact of depression on probability of achieving MPR threshold of 0.80.

In addition to examining the impact of depression on MPR levels, Figure 2 examines how comorbid depression affects the probability of achieving a threshold of at least 80%. These results are generally consistent with the results from the ordinary least squares regression which examine MPR as a continuous variable. Specifically, results from these multivariate logistic models indicate that patients who were depressed (based upon either diagnosis or diagnosis and receipt of antidepressant) were 45% less likely to achieve an MPR threshold of 80% (odds ratio (OR) 0.55; 95% confidence interval (CI) 0.42–0.74). An examination of the subset of patients with both MS and depression revealed that those treated with an antidepressant, irrespective of length of treatment, had no significant difference in the likelihood of achieving an MPR threshold of 80% (OR = 1.32; 95% CI 0.50–3.48). However, those treated with an antidepressant for at least 6 months were twice as likely to reach an MPR threshold of 80% with their DMT, compared to those treated with an antidepressant for less than 6 months (OR = 2.17; 95% CI 1.07–4.39).

### 3.4. Robustness of Results

As a test of the robustness of the results, alternative specifications were also examined. First, we constructed an alternative model that allowed for patient age, sex, region of residence, and ITT medication to be explicitly included in the multivariate analyses. Second, as discussed above, adherence was also proxied by the proportion of days covered. Third, to examine the impact of the relatively small sample when comparing those patients diagnosed with depression but untreated to those diagnosed with depression who received an antidepressant, the analysis also examined this subgroup without the reduction of sample size that is the result of matching the two cohorts; none of these modifications resulted in any significant changes in the results reported. 

### 3.5. Discussion

Focused on a population consisting entirely of patients with MS, this analysis was designed to determine the impact of comorbid depression on adherence to DMT. This study also sought to determine the impact of antidepressant therapy on adherence to DMT among patients with MS and comorbid depression. This analysis produced several noteworthy results.

First, patients diagnosed with comorbid depression were sicker than those without comorbid depression, as indicated by their likelihood of a higher score on the Charlson Comorbidity Index (see Table 1), as well as their greater likelihood of having a musculoskeletal limitation (60.09% versus 41.51%; *P* < 0.01) or any medical limitation (48.17% versus 31.42%; *P* < 0.01). This association between comorbid depression and a larger burden of illness is consistent with research on other disease states, research which has shown that patients with diabetes and comorbid depression have more complications relative to diabetic patients without depression [30] and that heart disease patients with comorbid depression have greater morbidity and mortality relative to their counterparts without depression [31–37].

Second, patients with MS and comorbid depression had, on average, a 10-point lower MPR and were approximately half (45%) as likely to reach a threshold MPR level of 80% relative to the matched cohort with MS without depression. The strong association between depression and difficulty with adherence was expected and was consistent with the findings of earlier research conducted among patients with MS [10, 13]. It also was consistent with research showing the negative effect of comorbid depression on adherence to therapy for other diseases, including hypertension [38], diabetes [10, 11], and heart disease [39, 40]. While previous research among patients with MS has shown comorbid depression to be associated with adherence difficulties [10, 13], our research expands the literature by quantifying the adherence gap between those MS patients with comorbid depression compared to those without depression.

Third, results from this analysis revealed no significant difference in adherence to DMT therapy when comparing patients diagnosed with depression that were not treated with an antidepressant to those both diagnosed and treated for depression. However, among patients with comorbid depression, those who received at least a 6-month supply of antidepressants had, on average, a 10-point higher MPR and were more than twice as likely to reach an MPR threshold of 80% relative to those who filled their antidepressant medication prescription for less than a 6-month period. Significantly, in our study, merely having a prescription for an antidepressant had no significant impact on DMT adherence. Instead, the positive impact of antidepressant therapy on DMT adherence was observed only among patients whose antidepressant therapy extended for at least 6 months. This finding supports previous research conducted among patients diagnosed with diabetes, which showed that antidepressant therapy improved adherence to diabetes treatment and medical outcomes [41]. However, caution must be taken when interpreting this finding since results are also consistent with the hypothesis that patients who are adherent to their antidepressant therapy are generally more compliant at taking their medications and hence, more likely to be adherent to their DMT as well.

### 3.6. Limitations

As with any research, the findings presented here should be interpreted within the context of the limitations of the study design. First, this analysis was conducted using an administrative claims database and included only patients with medical and outpatient prescription drug benefit coverage. The results, therefore, may not generalize well to other populations. Second, it is less rigorous to rely upon diagnostic codes rather than formal diagnostic assessment for identifying patients or for measuring patient general health and functional limitations. For example, our reliance on diagnostic claims and the criteria utilized for identifying patients with depression resulted in approximately 6% of our MS cohort with comorbid depression while previous research has shown that up to 50% of MS patients experience comorbid depression [14, 15]. Third, this analysis used MPRs and PDCs to measure medication adherence. Such measures are unable to identify actual drug-taking behavior or patient adherence to filling written prescriptions. Fourth, the database was unable to capture other information, such as patient personality type, caregiver support, and type of MS, that may also impact outcomes. Finally, this study focuses on a subset of individuals who were diagnosed with depression prior to receipt of a DMT for MS. The results, therefore, may not apply to individuals who are diagnosed with depression subsequent to initiation on treatment for MS. 

## 4. Conclusions

In conclusion, this study indicates that those with MS and comorbid depression are sicker and approximately only half as likely to be adherent to their disease-modifying therapy relative to patients with MS without comorbid depression. This analysis also shows were no significant difference in DMT adherence when comparing patients diagnosed with depression but not treated with an antidepressant to those both diagnosed and treated for depression. However, those with MS and comorbid depression who take antidepressants for at least 6 months are more than twice as likely to be adherent to their DMT relative to those with MS and comorbid depression who do not remain on antidepressant therapy for at least 6 months. Future work should focus on examining these results with alternative data sources and improving our understanding of the relationship between adherence to antidepressant therapy and adherence to DMT.

## Figures and Tables

**Table 1:** Patient characteristics: patients with MS Only compared to Patients with MS & Depression.

Characteristic	MS Only (*N* = 436)		MS & Depression (*N* = 436)		*χ* ^2^ statistic	*P* value
	*N*	%	*N*	%		
*Characteristics matched on*						
Age					N/A	N/A
<35	54	12.39	54	12.39		
35–44	107	24.54	107	24.54		
45–54	171	39.22	171	39.22		
55–64	95	21.79	95	21.79		
65+	9	2.06	9	2.06		
Sex					N/A	N/A
Male	55	12.61	55	12.61		
Female	381	87.39	381	87.39		
Region					N/A	N/A
Northeast	53	12.16	53	12.16		
North Central	124	28.44	124	28.44		
South	166	38.07	166	38.07		
West	93	21.10	93	21.10		
Unknown	1	0.23	1	0.23		
Year of index date					N/A	N/A
2004	66	15.14	66	15.14		
2005	109	25.00	109	25.00		
2006	109	25.00	109	25.00		
2007	129	29.59	129	29.59		
2008	23	5.28	23	5.28		
ITT DMT					N/A	N/A
Avonex	97	22.25	97	22.25		
Betaseron	51	11.70	51	11.70		
Copaxone	198	45.41	198	45.41		
Rebif	78	17.89	78	17.89		
Tysabri	12	2.75	12	2.75		

*Other characteristics*						
Insurance status						
Comprehensive	58	13.30	43	9.86	2.52	0.112
Health maintenance org	80	18.35	90	20.64	0.73	0.393
Point-of-service	99	22.71	58	13.30	13.06	<0.001
Preferred provider org	191	43.81	226	51.83	5.63	0.018
Other	8	1.83	19	4.36	4.63	0.032
Copayment with ITT drug	370	84.86	340	77.98	6.82	0.009
Mail order for ITT drug	145	33.26	119	27.29	3.67	0.055
Preperiod Charlson score						
0	331	75.92	271	61.16	19.31	<0.001
1-2	93	21.33	132	30.28	9.11	0.003
3+	12	2.75	33	7.57	10.33	<0.001
Preperiod disability proxies						
Muscoloskeletal	181	41.51	262	60.09	30.10	<0.001
Medical	137	31.42	210	48.17	25.51	<0.001

**Table 2:** Patient characteristics: MS patients diagnosed with depression but untreated compared to MS patients diagnosed with depression who received an antidepressant.

Characteristic	Dx Depression (*N* = 38)		Dx & Rx Depression (*N* = 38)		*χ* ^2^ statistic	*P* value
	*N*	%	*N*	%		
*Characteristics matched on*						
Age					N/A	N/A
<35	1	2.63	1	2.63		
35–44	8	21.04	8	21.04		
45–54	19	50.00	19	50.00		
55–64	10	26.32	10	26.32		
65+	0	0	0	0		
Sex					N/A	N/A
Male	2	5.26	2	5.26		
Female	36	94.74	36	94.74		
Region					N/A	N/A
Northeast	3	7.89	3	7.89		
North Central	13	34.21	13	34.21		
South	12	31.58	12	31.58		
West	10	26.32	10	26.32		
Unknown	0	0	0	0		
Year of index date					N/A	N/A
2004	6	15.79	6	15.79		
2005	11	28.95	11	28.95		
2006	10	26.32	10	26.32		
2007	10	26.32	10	26.32		
2008	1	2.63	1	2.63		
ITT DMT					N/A	N/A
Avonex	12	31.58	12	31.58		
Betaseron	2	5.26	2	5.26		
Copaxone	19	50.00	19	50.00		
Rebif	4	10.53	4	10.53		
Tysabri	1	2.63	1	2.63		

*Other characteristics*						
Insurance status						
Comprehensive	6	15.79	4	13.16	0.11	0.744
Health maintenance org	5	13.16	9	23.68	1.40	0.236
Point-of-service	6	15.79	7	18.42	0.93	0.761
Preferred provider org	20	52.63	16	42.11	0.84	0.358
Other	1	2.63	1	2.63	N/A	N/A
Copayment with ITT drug	29	76.32	29	76.32	N/A	N/A
Mail order for ITT drug	13	34.21	10	26.32	0.56	0.454
Preperiod Charlson score						
0	23	60.53	21	55.26	0.22	0.642
1-2	11	28.95	15	39.47	0.93	0.334
3+	4	10.53	2	5.26	0.72	0.395
Preperiod disability proxies						
Muscoloskeletal	25	65.79	24	63.16	0.06	0.811
Medical	16	42.11	18	47.37	0.22	0.645

**Table 3:** Patient characteristics: MS patients treated with antidepressants for at least 6 months compared to MS patients treated with antidepressants for less than 6 months.

Characteristic	Treated less than guidelines (*N* = 71)		Treated according to guidelines (*N* = 71)		*χ* ^2^ statistic	*P* value
	*N*	%	*N*	%		
*Characteristics matched on*						
Age					N/A	N/A
<35	5	7.04	5	7.04		
35–44	17	23.94	17	23.94		
45–54	32	45.07	32	45.07		
55–64	17	23.94	17	23.94		
65+	0	0	0	0		
Sex					N/A	N/A
Male	1	1.41	1	1.41		
Female	70	98.59	70	98.59		
Region					N/A	N/A
Northeast	5	7.04	5	7.04		
North Central	21	29.58	21	29.58		
South	30	42.25	30	42.25		
West	15	21.13	15	21.13		
Unknown	0	0	0	0		
Year of index date					N/A	N/A
2004	6	8.45	6	8.45		
2005	17	23.94	17	23.94		
2006	15	21.13	15	21.13		
2007	28	39.44	28	39.44		
2008	5	7.04	5	7.04		
ITT DMT					N/A	N/A
Avonex	11	15.49	11	15.49		
Betaseron	3	4.23	3	4.23		
Copaxone	44	61.97	44	61.97		
Rebif	13	18.31	13	18.31		
Tysabri	0	9	0	9		

*Other characteristics*						
Insurance status						
Comprehensive	6	8.45	6	8.45	N/A	N/A
Health maintenance org	16	22.54	10	14.08	1.70	0.193
Point-of-service	10	14.08	6	8.45	1.13	0.288
Preferred provider org	39	54.93	45	63.38	1.05	0.306
Other	0	0	4	5.63	4.12	0.043
Copayment with ITT drug	61	85.92	54	76.06	2.24	0.134
Mail order for ITT drug	23	32.39	22	30.99	0.03	0.857
Preperiod Charlson score						
0	45	63.38	40	56.34	0.73	0.392
1-2	20	28.17	26	36.62	1.16	0.282
3+	6	8.45	5	7.04	0.09	0.754
Preperiod disability proxies						
Muscoloskeletal	46	64.79	48	67.61	0.13	0.723
Medical	35	49.30	42	59.15	1.39	0.238

**Table 4:** Ordinary least squares regression results dependent variable: MPR.

Variable	MS Only versus MS & Depression		Dx Depression versus Dx & Rx Depression		Rx Depression- ≥6 Mo Antidepressants versus <6 Mo	
	Coeff	*P* value	Coeff	*P* value	Coeff	*P* value
Intercept	0.71	<0.001	0.64	0.047	0.47	<0.001
Insurance type^a^						
HMO	−0.10	0.022	−0.14	0.509	-0.16	0.200
POS	−0.02	0.665	−0.07	0.731	0.02	0.901
PPO	0.01	0.890	0.12	0.491	0.02	0.860
Other	−0.002	0.974	−0.27	0.523	0.08	0.693
Medication dispensing						
Copayment	0.06	0.047	−0.06	0.680	0.10	0.213
Mail order	0.05	0.051	0.02	0.909	0.08	0.216
Preperiod Charlson score^b^						
1-2	0.01	0.738	0.09	0.521	0.06	0.379
3+	−0.04	0.467	0.04	0.853	0.09	0.422
Preperiod ADL proxies						
Musculoskeletal	0.02	0.440	−0.03	0.828	0.06	0.294
Medical	0.02	0.448	0.10	0.419	−0.01	0.889
Preperiod depression						
Any	−0.10^c^	<0.001	—	—	—	—
Dx & Rx	—	—	0.07^d^	0.518	—	—
Rx for at least 6 months	—	—	—	—	0.12^e^	0.049
Adjusted *R* ^2^	0.05		0.08		0.06	

^a^Reference group: Comprehensive insurance.

^b^Reference group: Charlson score of 0.

^c^Reference group: patients with MS and no diagnosis of depression or receipt of an antidepressant.

^d^Reference group: patients diagnosed with depression who did not receive an antidepressant.

^e^Reference group: patients diagnosed with depression who were treated with an antidepressant for less than 6 months.
